# Concurrent splenic infarction and pulmonary embolism as the initial presentation of high-grade follicular lymphoma: a case report

**DOI:** 10.3389/fonc.2026.1987312

**Published:** 2026-10-29

**Authors:** Qijiang Zhao, Chaoli Ding, Suping Wu, Yumei Zeng

**Affiliations:** Department of General Medicine and Cardiopulmonary Rehabilitation, The Affiliated Hospital of Shaoxing University, Shaoxing, Zhejiang, China

**Keywords:** case report, follicular lymphoma, hypercoagulability, pulmonary embolism, splenic infarction

## Abstract

**Background:**

Follicular lymphoma (FL) is a common indolent non-Hodgkin lymphoma, reports of concurrent splenic infarction and pulmonary embolism as the initial complication are relatively uncommon. We report a case of high-grade follicular lymphoma presenting with pulmonary embolism and splenic infarction as prominent clinical features, aiming to improve the recognition of atypical manifestations of this disease.

**Case presentation:**

The patient was presented with long-term multiple enlarged lymph nodes throughout the body. Laboratory tests showed elevated D-dimer and β2-microglobulin levels, as well as thrombocytopenia. Imaging studies demonstrated multiple lymphadenopathies, marked splenomegaly with increased metabolic activity, and diffuse bone marrow hypermetabolism.

**Results:**

The patient underwent lymph node biopsy of the left cervical and right axillary regions, histopathological examination and immunohistochemistry of the lymph node biopsy confirmed the diagnosis of high-grade follicular lymphoma. Bone marrow examination revealed bone marrow infiltration. The patient received R-CDOP regimen chemotherapy, at the time of discharge, the patient’s condition was relatively stable.

**Conclusion:**

This case suggests that in patients with unexplained splenomegaly complicated by thrombotic events and long-standing lymphadenopathy, a high index of suspicion for underlying follicular lymphoma or other lymphoproliferative disorders should be maintained. Standardized pathological and imaging evaluations are essential to guide precise treatment.

## Introduction

Splenic infarction and pulmonary embolism are distinct thrombotic events. They present with different clinical features. However, they share a common pathophysiological core mechanism: hypercoagulability. When both occur simultaneously, and cardioembolic sources or traditional VTE risk factors are absent, clinicians should suspect an underlying systemic hypercoagulable state. This condition often indicates an underlying hematological disorder (1, 2).

Splenic infarction is a relatively rare acute abdominal condition. Its main etiologies include cardioembolism, splenic arterial thrombosis, and hematological diseases. In oncology, splenic infarction is more commonly observed in myeloproliferative neoplasms and leukemias. Although splenic infarction can occur in lymphoma, such cases are exceedingly rare (3, 4). In contrast, in patients with non-Hodgkin lymphoma, the incidence of VTE is approximately 6.5%, which is significantly higher than that in the general population (5). The occurrence of VTE is closely associated with tumor burden, inflammatory response, and coagulation system activation. Therefore, unexplained splenic infarction and pulmonary embolism should be regarded as a vital sign of systemic hypercoagulability. This is especially true when both events coexist.

We herein report a case of grade IIIa follicular lymphoma in a patient whose initial clinical manifestations were pulmonary embolism and splenic infarction. The diagnosis was ultimately confirmed by lymph node biopsy. What distinguishes this case is not the coexistence of thrombosis and lymphoma, which is well recognized, but the fact that splenic infarction and pulmonary embolism occurred together as the initial presentation, with splenic infarction developing in the immediate post-biopsy period before the diagnosis was confirmed. Splenic infarction is exceedingly rare in lymphoma, and this sequence has rarely been reported.

## Case report

A 63-year-old male patient had a past medical history of nasal cyst and left lower extremity varicose vein surgery. He reported multiple enlarged lymph nodes throughout the body for several years, which had not been specifically evaluated or treated. In February 2026, the patient presented to an outside hospital due to pulmonary embolism. During the evaluation, computed tomography pulmonary angiography (CTA) incidentally revealed marked splenomegaly (**Figures 1A, B**).

**Figure 1:**
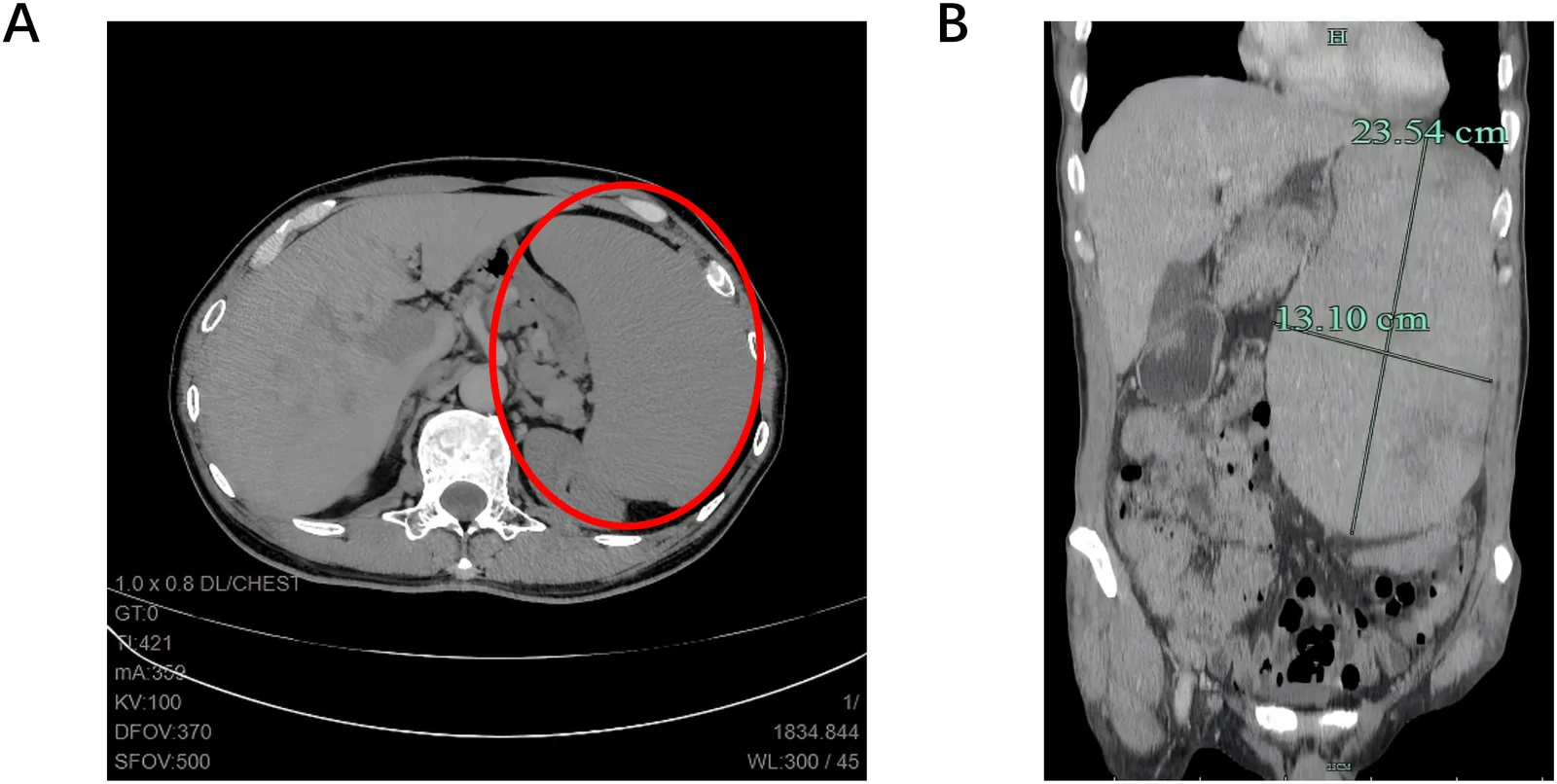
Marked splenomegaly on CT. **(A)** Axial contrast-enhanced CT showing marked splenomegaly (red circle). **(B)** Coronal contrast-enhanced CT showing the enlarged spleen, with a longitudinal diameter of 23.54 cm and a transverse diameter of 13.10 cm.

To clarify the etiology of the lymphadenopathy and splenomegaly, the patient was admitted to our department on March 30, 2026. On admission, the patient was conscious and cooperative. Vital signs were stable: temperature 36.4 °C, pulse 95 beats/min, respiratory rate 20 breaths/min, blood pressure 153/75 mmHg. Enlarged superficial lymph nodes were palpable in the submandibular, axillary, and inguinal regions. Cardiopulmonary examination was unremarkable: clear breath sounds without rales, regular heart rhythm at 95 beats/min without murmurs. Abdominal examination revealed a large, hard, palpable mass, and the spleen was palpable and enlarged; there was no tenderness or rebound tenderness. No lower-limb edema. Neurological examination was normal. Laboratory tests showed hypersensitive C-reactive protein (hs-CRP) at 25 mg/L, D-dimer at 5.07 μg/mL FEU, platelet count at 98 × 10^9^/L, and β2-microglobulin at 18.72 mg/L. Serological testing was positive for hepatitis B surface antigen and core antibody, and Epstein-Barr virus (EBV) DNA load was elevated (7.12 × 10³ IU/mL).

Ultrasonography revealed multiple enlarged lymph nodes in the bilateral cervical, axillary, and inguinal regions with ill-defined corticomedullary differentiation (**Supplementary Figures 1A–C**). Contrast-enhanced abdominal CT demonstrated multiple lymph nodes in the hepatic hilum, retroperitoneum, and inguinal regions, marked splenomegaly with focal hypoenhancing areas suggestive of splenic infarction, and portal vein widening with splenic vein and gastric fundal varices (**Figures 2A–C**). Computed tomography pulmonary angiography (CTPA) revealed a filling defect in the right pulmonary artery (red arrow), consistent with pulmonary embolism (**Supplementary Figure 2A**). PET/CT showed increased FDG uptake in multiple lymph nodes above and below the diaphragm (SUVmax 14.28), increased splenic uptake (SUVmax 12.35), and diffuse bone marrow hypermetabolism in the axial skeleton and parts of the appendicular skeleton (SUVmax 21.41) (**Figure 2D**).

**Figure 2:**
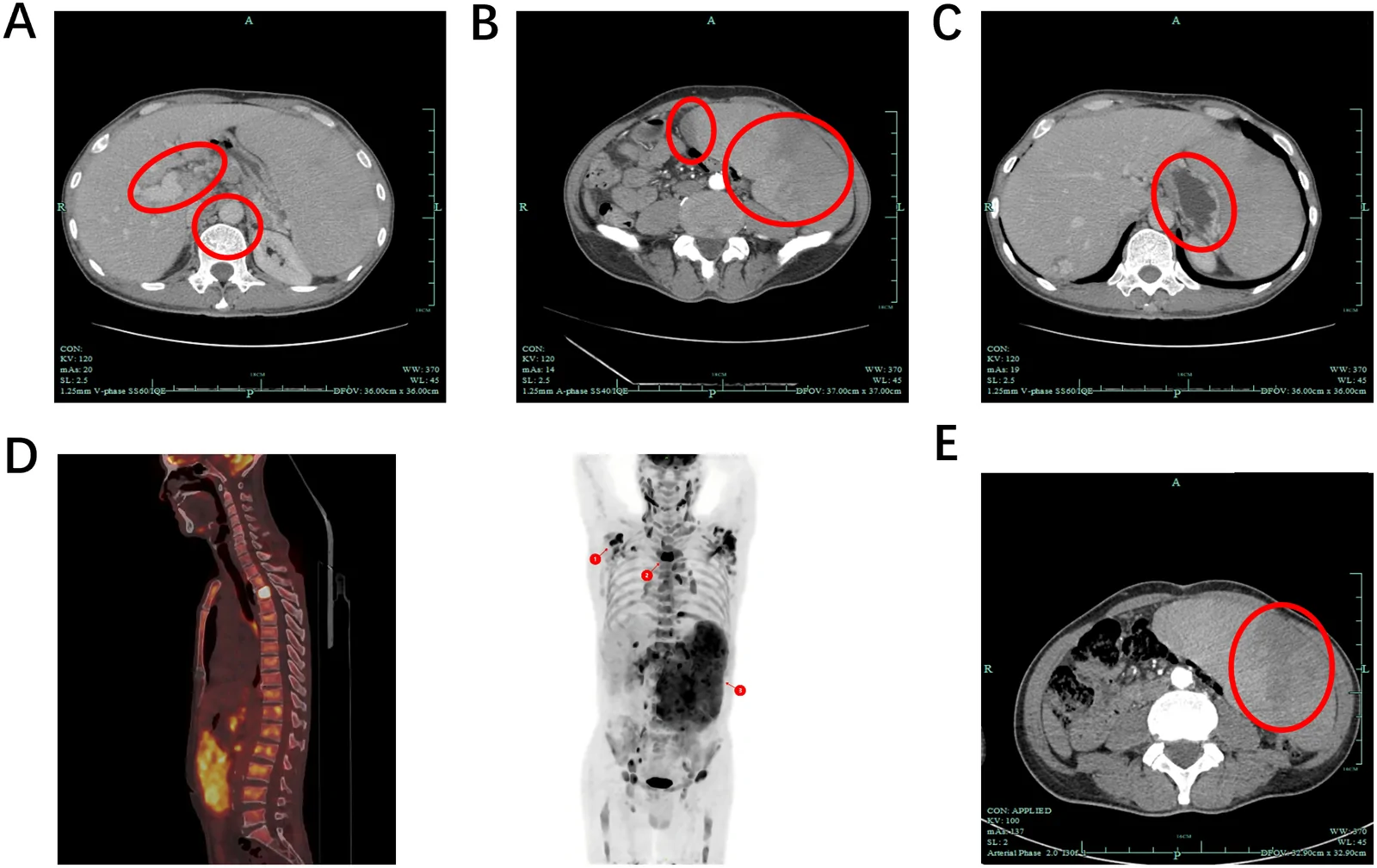
Contrast-enhanced abdominal CT and PET/CT findings. **(A)** Multiple enlarged lymph nodes and portal vein widening are visible in the hepatic hilum and retroperitoneum. **(B)** CT image reveals heterogeneous enhancement of the spleen with a wedge-shaped hypodense lesion, consistent with splenic infarction; **(C)** gastric fundal varices are noted; **(D)** PET/CT showed increased FDG uptake in multiple lymph nodes above and below the diaphragm, increased splenic uptake, and diffuse bone marrow hypermetabolism in the axial skeleton and parts of the appendicular skeleton, abnormal sites are indicated; **(E)** contrast-enhanced CT confirmed acute splenic infarction.

To further establish a definitive diagnosis, the patient underwent lymph node biopsy of the left cervical and right axillary regions on April 3, 2026. On the day following the procedure, the patient experienced acute left-sided abdominal pain; contrast-enhanced CT confirmed acute splenic infarction (**Figure 2E**). Symptomatic supportive treatments including anticoagulation and analgesia were administered accordingly.

Histopathological examination and immunohistochemical staining of the lymph node biopsy revealed the following results: CD20 (+), CD10 (+), BCL-6 (+), BCL-2 (+), CD21 (+), Ki-67 (approximately 60% in hot spots), CD3 (T cells+), CD5 (T cells+), Cyclin D1 (–), CD30 (–), Mum-1 (focal+), and EBER (–). The pathological diagnosis was non-Hodgkin lymphoma, consistent with high-grade follicular lymphoma (WHO grade IIIa).

Bone marrow aspiration revealed decreased nucleated cells with a relatively increased lymphocyte proportion (32.3%). Bone marrow biopsy demonstrated that abnormal B lymphocytes accounted for approximately 50% of cellularity, with immunohistochemical staining showing CD20 (+), CD10 (partial+), BCL-6 (–), and Cyclin D1 (–). Flow cytometry detected a mature B-cell clone with partial CD10 positivity and scattered CD5 positivity, consistent with the immunophenotype of follicular lymphoma.

Integrating the lymph node biopsy results, PET/CT staging, and bone marrow findings, the patient was definitively diagnosed with follicular lymphoma (WHO grade IIIa, stage IV B, FLIPI score 4, high-risk group), with secondary splenic infarction. The patient was identified as a hepatitis B virus carrier with active infection. Prior to chemotherapy, entecavir was given for HBV prophylaxis, and acyclovir for herpes simplex virus and varicella-zoster virus prophylaxis during rituximab-based therapy. After discussion with the patient, the R-CDOP regimen was initiated, consisting of rituximab 0.6 g on day 0; pegylated liposomal doxorubicin 17.5 mg on days 1–2; vindesine 1.75 mg on days 1–2; cyclophosphamide 0.35 g on days 1–2; and dexamethasone 6 mg on days 1–5. Supportive treatments including liver protection, gastric mucosal protection, uric acid reduction, and anti-infection prophylaxis with compound sulfamethoxazole were also provided.

The patient demonstrated a favorable response to treatment. Follow-up computed tomography pulmonary angiography (CTA) on May 5, 2026, revealed a reduction in pulmonary embolic lesions (**Figure 3A**), and CTPA showed no residual filling defect in the pulmonary arteries, indicating resolution of the previously identified pulmonary embolism (**Supplementary Figure 2B**). Contrast-enhanced CT on May 28, 2026, showed a decrease in splenic size (**Figure 3B**). PET/CT on July 21, 2026, demonstrated normalization of metabolic activity (**Figure 4A**). Bone marrow aspiration on July 22, 2026, revealed post-treatment normocellular marrow with trilineage maturation, and no significant phenotypic abnormalities or aggregation of lymphocytes were observed. Flow cytometry on the same day detected no abnormal B-cell population. At the last follow-up on October 9, 2026, the patient remained in good condition with no evidence of disease recurrence.

**Figure 3:**
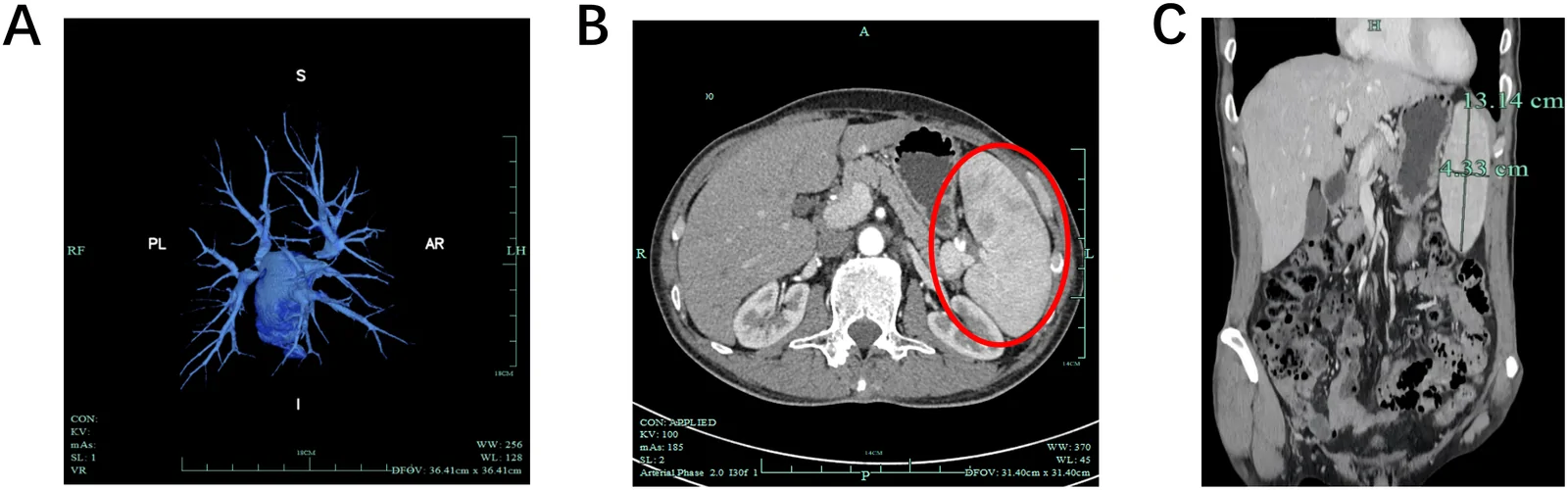
Follow-up imaging showing treatment response. **(A)** Computed tomography pulmonary angiography (CTA) demonstrated marked regression of pulmonary emboli. **(B)** Contrast-enhanced abdominal CT showed a substantial decrease in splenic size. **(C)** Contrast-enhanced CT showed a substantial decrease in splenic size.

**Figure 4:**
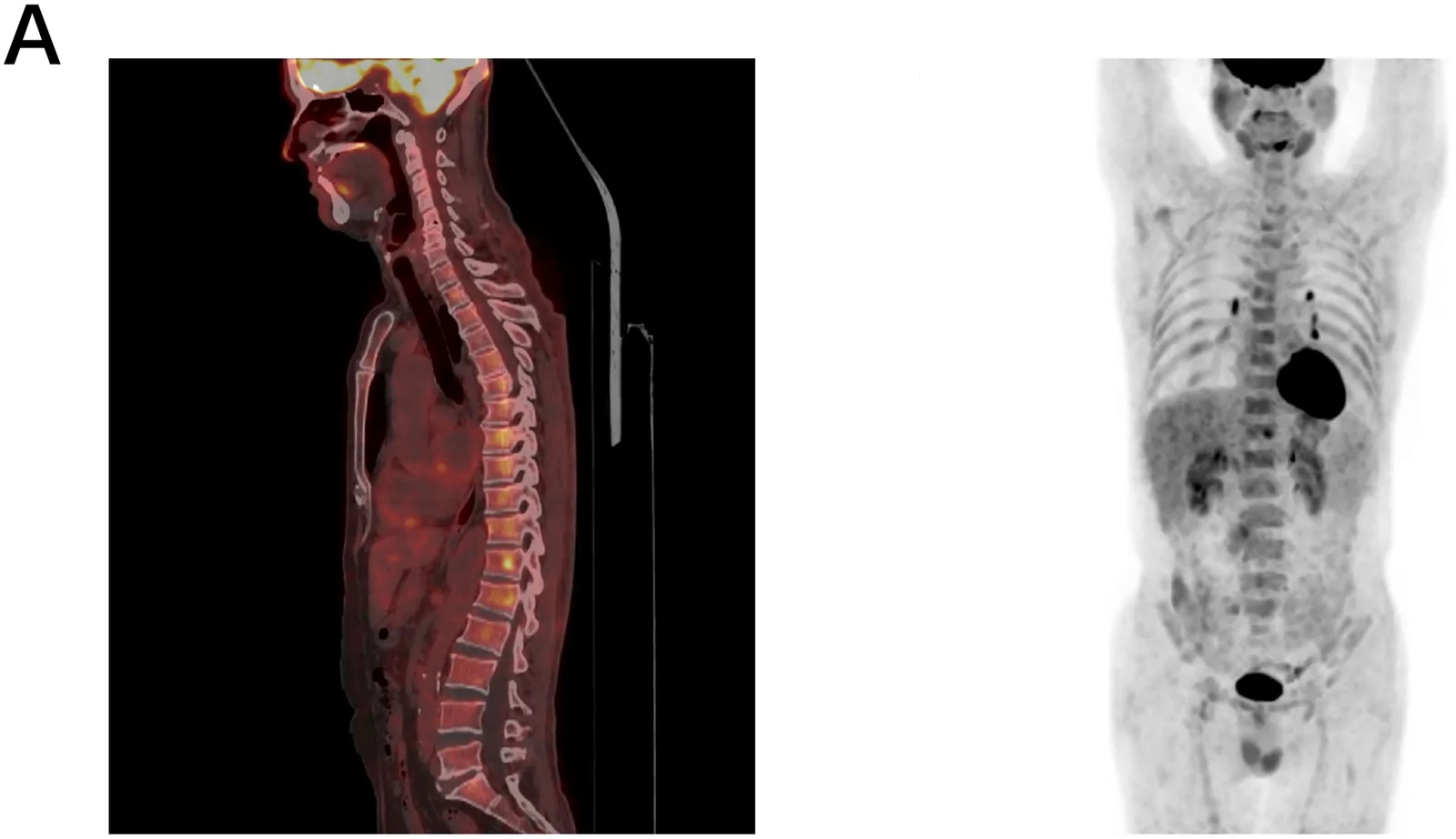
Follow-up PET/CT. **(A)** PET/CT demonstrated normalization of metabolic activity.

The key clinical events and treatment course of this patient are summarized in **Table 1**.

**Table 1:** Chronological summary of clinical events, diagnostic workup, and treatment response.

Time	Event	Key findings	Intervention	Outcome
Years prior	Asymptomatic lymphadenopathy	Multiple enlarged nodes	None	Stable
Day 0	Admission	Firm, mobile nodes; D-dimer 5.07, PLT 98	Examination initiated	None
Day 4	Lymph node biopsy	None	Diagnostic biopsy	None
Day 5	Acute left abdominal pain	CT: acute splenic infarction	Anticoagulation, analgesia	Symptom relief
Day 7	Pathology result	WHO grade IIIa FL (Ki-67 60%)	R-CDOP initiated	None
Week 5	Follow-up CTPA	Pulmonary emboli reduced	Continue treatment	Favorable
Week 16	Follow-up PET/CT	Metabolic normalization	None	Remission

## Discussion

We report a case of grade IIIa follicular lymphoma presenting with pulmonary embolism and splenic infarction as prominent clinical manifestations, the diagnostic and therapeutic process of this case carries significant clinical value. The diagnostic clue in this case originated from an incidental finding: splenomegaly discovered during CTA performed for pulmonary embolism. The patient had a history of long-standing, asymptomatic lymphadenopathy for several years, which had not been specifically evaluated. This underscores that for patients with long-standing, asymptomatic lymphadenopathy, even in the absence of subjective symptoms, systematic evaluation is warranted to enable early identification of indolent lymphoma. While venous thromboembolism is a recognized complication of lymphoma, splenic infarction is far less common and is usually reported in myeloproliferative neoplasms or in isolated case reports. The simultaneous occurrence of both events before diagnosis, and the subsequent need to balance anticoagulation against post-biopsy bleeding risk, make this case instructive.

In this patient, the diagnosis was made step by step. Initial laboratory tests showed elevated D-dimer and β2-microglobulin, and a low platelet count, which suggested a possible hematological disease. Ultrasound and contrast-enhanced CT showed enlarged lymph nodes in several regions and marked splenomegaly with focal hypoenhancing areas, suggesting splenic infarction. PET/CT showed increased FDG uptake in multiple lymph nodes, the spleen, and bone marrow, which helped guide biopsy and staging. The final diagnosis was based on lymph node biopsy with histopathology and immunohistochemistry, which confirmed high-grade follicular lymphoma (WHO grade IIIa). Bone marrow aspiration, biopsy, and flow cytometry showed bone marrow involvement and supported the immunophenotype. Together, these tests allowed accurate diagnosis, staging, and risk stratification. These diagnostic findings are best understood in the context of the biological features of follicular lymphoma. Follicular lymphoma (FL) is an indolent non-Hodgkin lymphoma originating from germinal center B cells, characterized by marked clinical heterogeneity (6). According to the World Health Organization (WHO) classification, FL is graded from 1 to 3 based on the number of centroblasts per high-power field, with grade 3 further subdivided into 3a and 3b (7). Unlike indolent grades 1–2, grade 3 FL is more aggressive. Grade 3a shares clinicopathological features with grade 3b and diffuse large B-cell lymphoma, and some cases pursue an aggressive course (8). The diagnosis of FL relies heavily on histopathological and immunohistochemical examination, with a classic immunophenotype of CD20 (+), CD10 (+), BCL-6 (+), and BCL-2 (+) (6). However, pathological grading and diagnosis of FL face multiple challenges. Inter-observer reproducibility of grading criteria is suboptimal, immunophenotypic discrepancies may exist between biopsies from different sites, and some FL cases may exhibit atypical immunophenotypes, increasing the difficulty of differential diagnosis (9, 10). Our case exemplifies this aggressive subset: the patient exhibited a Ki-67 proliferation index of up to 60%, PET/CT findings of multiorgan extranodal involvement (spleen, bone marrow, nasopharynx), and a FLIPI score of 4, all of which indicate poor prognosis and high-risk stratification. Although FL typically presents with painless lymphadenopathy, some cases may present due to compression by bulky lymphadenopathy, marked splenomegaly, or systemic symptoms. Splenomegaly is a common manifestation of FL; however, reports of splenic infarction as the initial complication are exceedingly rare (11). Our case represents this rare presentation: acute splenic infarction occurred after lymph node biopsy in a patient with concurrent massive splenomegaly and pulmonary embolism. Splenic infarction in this patient was likely multifactorial. Tumor-associated hypercoagulability, reflected by persistently elevated D-dimer, was probably the main mechanism, but massive splenomegaly and altered splenic/portal hemodynamics, including portal vein widening and splenic vein and gastric fundal varices, may also have contributed. The infarction occurred on the day after biopsy, suggesting that the procedure may have further compromised an already vulnerable spleen. The coexistence of splenic infarction and pulmonary embolism, two thrombotic events in the same patient, posed a clinical dilemma: balancing anticoagulation against bleeding risk from the recent biopsy. The occurrence of both events underscores the need for multidisciplinary management of competing thrombotic and hemorrhagic risks. VTE risk in lymphoma is strongly subtype-dependent, with an incidence of approximately 1.9% in indolent lymphomas and 8–11% in aggressive subtypes. Grade 3A follicular lymphoma is conventionally regarded as indolent, so the expected thrombotic risk would be low. This contrast further supports the atypical nature of the present case.

Several difficulties were encountered during diagnosis. First, the patient had enlarged lymph nodes for years but had never been evaluated, which delayed recognition of the underlying lymphoma. Second, the initial presentation was mainly thrombotic events, pulmonary embolism and splenic infarction, rather than typical lymphoma symptoms, which drew attention away from the hematological disease; in particular, splenic infarction is extremely rare in lymphoma and can easily be attributed to other causes such as cardioembolism. Third, the presence of both splenic infarction and pulmonary embolism made treatment difficult, because anticoagulation had to be balanced against bleeding risk after recent biopsy. Therefore, anticoagulation was given cautiously after weighing the bleeding risk from the recent biopsy, together with analgesia, and the patient’s symptoms were relieved. These points highlight the need for a high suspicion, standard pathological and imaging evaluation, and multidisciplinary management in similar cases. In summary, this case highlights the diagnostic challenges of high-grade FL with thrombotic events. Unexplained splenomegaly, VTE, and lymphadenopathy should prompt evaluation for lymphoproliferative disorders. Management requires balancing thrombosis and bleeding risks, especially when biopsy is needed. For HBV-positive patients, antiviral prophylaxis is essential before and during rituximab-based therapy. Recognition of these atypical presentations may facilitate earlier diagnosis

## Patient’s perspective

I’d had swollen lymph nodes for years. I didn’t think much of it. They didn’t hurt, so I just left them alone. Then I ended up in the hospital with a clot in my lungs, and that’s when they found my spleen was enlarged too. They said a blood vessel in my spleen was blocked. I was worried about the blood thinners and the wound. The chemotherapy was hard. But the scans kept looking better, and that helped. I’m home now, doing okay.

## Conclusion

This case reports a high-grade follicular lymphoma presenting with prominent manifestations of pulmonary embolism, massive splenomegaly, splenic infarction, and multiregional lymphadenopathy. Its clinical course reveals that a subset of follicular lymphoma may exhibit aggressive behavior and be accompanied by complex complications. This case emphasizes the necessity of standardized pathological and imaging evaluations for unexplained splenomegaly and lymphadenopathy to guide precision treatment. Furthermore, standardized antiviral prophylaxis is of paramount importance for lymphoma patients with concurrent hepatitis B virus infection.

## Data Availability

The original contributions presented in the study are included in the article/**Supplementary Material**. Further inquiries can be directed to the corresponding authors.

## Supplementary material

Supplementary Figure 1 Ultrasonographic findings of lymphadenopathy. **(A–C)** Ultrasound images of the cervical **(A)**, axillary **(B),** and inguinal **(C)** regions show multiple enlarged lymph nodes with loss of normal hilar architecture and ill-defined corticomedullary differentiation, suggestive of lymphomatous involvement.

Supplementary Figure 2 Follow-up CTPA after treatment. **(A)** Pretreatment CTPA showing a filling defect in the right pulmonary artery (red arrow), consistent with pulmonary embolism. **(B)** Post-treatment follow-up CTPA showing resolution of the pulmonary embolism, with no residual filling defect in the pulmonary arteries.
